# GeneToCN: an alignment-free method for gene copy number estimation directly from next-generation sequencing reads

**DOI:** 10.1038/s41598-023-44636-z

**Published:** 2023-10-18

**Authors:** Fanny-Dhelia Pajuste, Maido Remm

**Affiliations:** Institute of Molecular and Cell Biology, University of Tartu, 23 Riia Str., 51010 Tartu, Estonia

**Keywords:** Bioinformatics, Gene duplication

## Abstract

Genomes exhibit large regions with segmental copy number variation, many of which include entire genes and are multiallelic. We have developed a computational method GeneToCN that counts the frequencies of gene-specific *k*-mers in FASTQ files and uses this information to infer copy number of the gene. We validated the copy number predictions for amylase genes (AMY1, AMY2A, AMY2B) using experimental data from digital droplet PCR (ddPCR) on 39 individuals and observed a strong correlation (*R* = 0.99) between GeneToCN predictions and experimentally determined copy numbers. An additional validation on FCGR3 genes showed a higher concordance for FCGR3A compared to two other methods, but reduced accuracy for FCGR3B. We further tested the method on three different genomic regions (SMN, NPY4R, and LPA Kringle IV-2 domain). Predicted copy number distributions of these genes in a set of 500 individuals from the Estonian Biobank were in good agreement with the previously published studies. In addition, we investigated the possibility to use GeneToCN on sequencing data generated by different technologies by comparing copy number predictions from Illumina, PacBio, and Oxford Nanopore data of the same sample. Despite the differences in variability of *k*-mer frequencies, all three sequencing technologies give similar predictions with GeneToCN.

## Introduction

Copy number variation (CNV) is a type of structural variation ranging from 50 to several million base pairs (bp)^1–5^. It is an unbalanced variation where a segment of the human genome can be deleted, duplicated, or repeated multiple times and the number of repeats varies between individuals. Around 4.8–9.5% of the human genome contributes to CNVs^6^, a larger proportion than to SNVs, which may be associated with phenotypic traits including susceptibility to complex diseases^7–9^ especially when the copy number variation overlaps a gene region^10^. In this work, we will focus on copy number estimation of repeated genes or functional domains.

The detection of copy number differences requires a special computational approach, different from SNV analysis. The most common methods for copy number estimation from the WGS data use read depth (RD) and/or paired-end mapping (PEM)^11–16^ algorithms associated with custom-made statistical models for copy number detection. PEM-based methods map both paired ends to the reference genome and detect copy number change when the distance of mapped reads is significantly different from the mean insert size of the fragments. For this reason, PEM-based approaches cannot detect long CNVs and are not suitable for evaluating the exact copy number. Methods based on read depth use the depth of coverage information from reads mapped to the reference genome. RD-based methods can detect larger CNVs and may be used with paired-end reads as well as single-end reads. These methods can also estimate more precise copy numbers. For example, AMYCNE^17^ is an RD method that has demonstrated its ability to accurately estimate higher copy numbers through validation on amylase genes. However, the accuracy may be low when estimating exact copy numbers, especially when the number of repeats is high. In addition, both approaches depend on read mapping which is time-consuming and often unreliable in complex and repetitive genomic regions. Furthermore, many methods only work using cohort data, unable to estimate copy numbers for single individuals or for a small set of samples.

An alternative approach is to use an alignment-free analysis that is based on counting and analyzing the frequencies of *k*-mers in individual genomes. *K*-mers (small substrings of DNA with length* k*) have been used for different purposes in genome analysis to efficiently handle huge amounts of genomic data^18–20^. Alignment-free methods do not require read alignment or mapping thus allowing fast and reliable genotyping of known variants^21, 22^, discovering novel variants^23^ and genotyping polymorphic Alu-elements^24^. Only a handful of fully alignment-free methods have been created for estimating copy number variation of gene regions. For example, a general alignment-free CNV detection software QuicK-mer2, which is also able to handle gene regions, has recently been published^25^. However, this software is paralog-specific and has difficulty handling cases where a gene or region has multiple copies in the reference genome.

In this study, we propose a novel alignment-free method GeneToCN for targeted copy number estimation of copy-variable genes. We pay special attention to the selection of robust and reliable *k*-mers in gene regions. Our approach allows estimating copy numbers for individual samples without the requirement of cohort data. We demonstrate our method's accuracy on the amylase gene family and FCGR3 genes as well as general useability on three other gene regions (NPY4R, SMN, and LPA Kringle IV type 2 domain).

## Results

### Method for alignment-free gene copy number estimation

**Figure 1:**
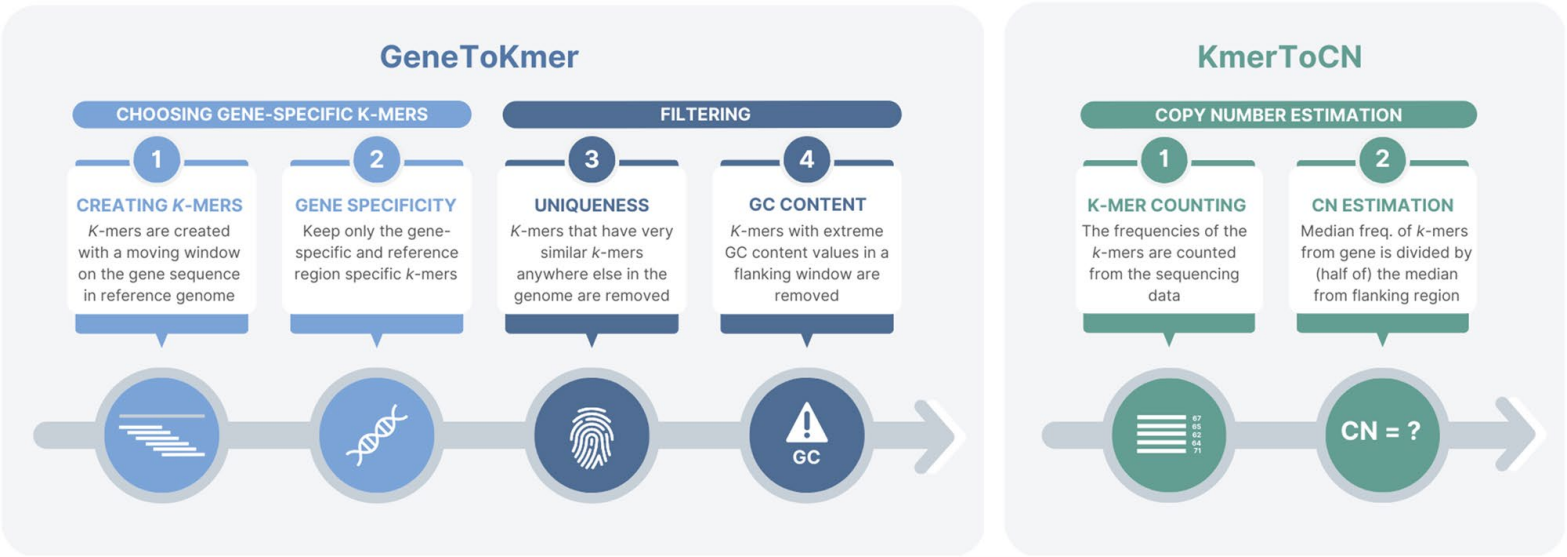
Overview of the method.

The working principle of the GeneToCN method is the following. First, a custom database is created consisting of carefully selected *k*-mers a) from a gene region and b) from the flanking regions of the same gene. The flanking regions are used to estimate the local depth of coverage (DOC), which is used as a reference in copy number estimation. The choice of representative *k*-mers for each gene is a crucial step of our method. To select the most robust and reliable set of reference *k*-mers, we apply several filters based on their uniqueness in the reference genome and their GC-content (described in Methods). The *k*-mer selection process is automated with the GeneToKmer script (Fig. 1).

Copy number estimation in each studied individual starts with counting the frequencies of the selected gene-specific *k*-mers directly from the raw sequencing reads of this individual. The copy number of each gene is calculated by dividing the median frequency of gene-region *k*-mers by the median frequency of flanking-region *k*-mers and multiplying by 2 (the ploidy of the human genome). The resulting copy number is decimal, but it can be rounded to the nearest integer if an integer copy number is preferred/required for interpretation. In this article, we use decimal numbers for correlation analysis and integers for concordance analysis.

Our method has a unique approach for handling regions in the reference genome that have multiple copies. For example, AMY1 is present in 3 copies in the reference. Unlike other methods that generally estimate the copy number separately for each of these copies, our GeneToKmer script has the flexibility to either treat them separately or to define all 3 copies as a single gene. In the first case, we use the *k*-mers specific to each different copy, whereas in the latter case, we use only k-mers that are present in all 3 copies. By avoiding the use of *k*-mers that may be variable due to recent mutations and are not present in all copies of a given gene, we can improve the accuracy of copy number predictions.

### Copy number estimation in AMY1, AMY2A, and AMY2B gene regions

**Figure 2:**
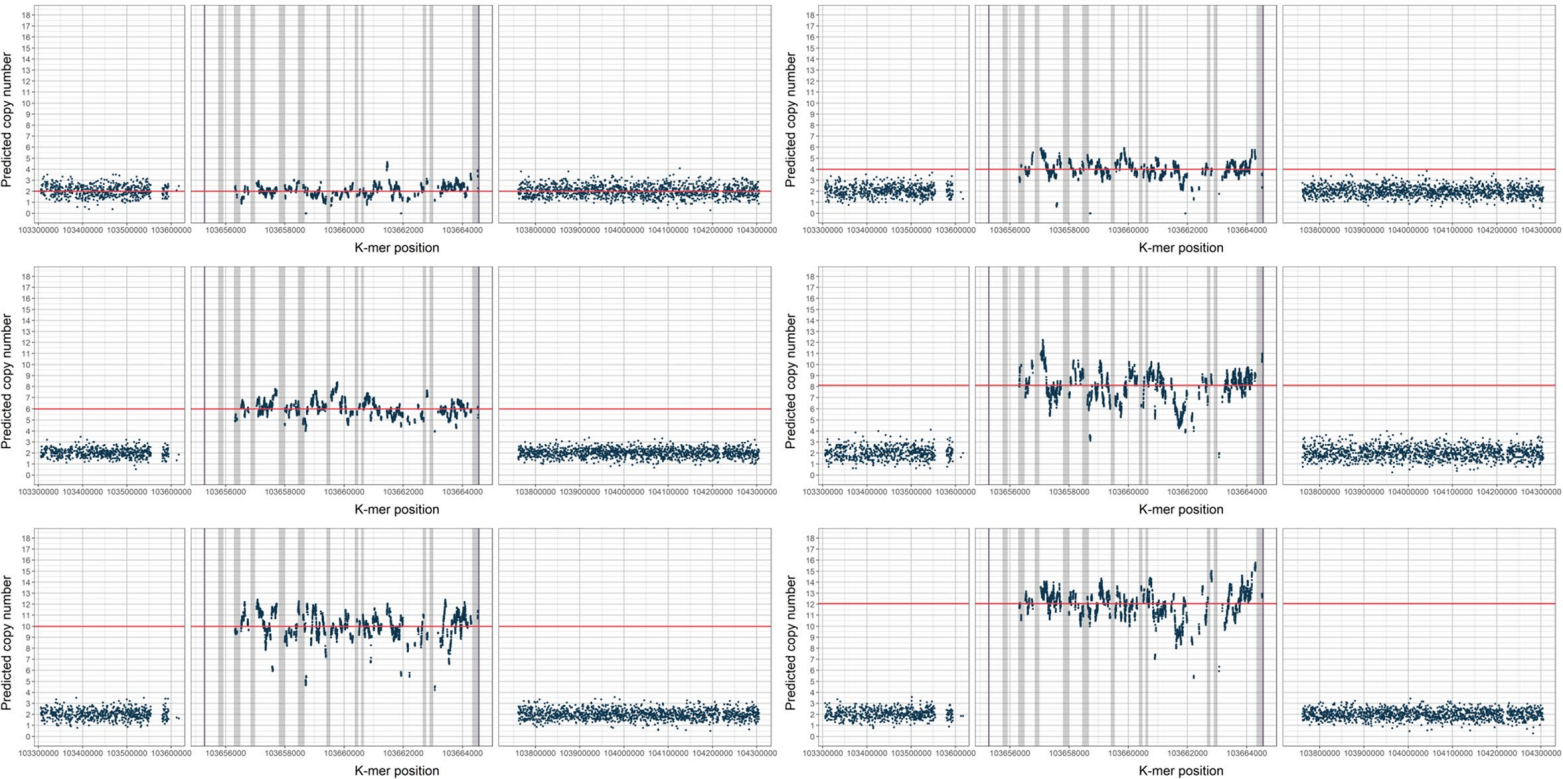
Normalized *k*-mer frequencies (*y*-axis) in different individuals, predicted to have 2, 4, 6, 8, 10, and 12 copies of the AMY1 gene. The *x*-axis shows the *k*-mer locations on chromosome 1. The horizontal red line marks the copy number estimated by GeneToCN. Each panel shows a 5'-flanking region, a zoomed-in AMY1 gene region, and a 3'-flanking region. Exon regions are shown in grey.

First, we investigated the performance of the GeneToCN method using the well-studied alpha-amylase gene family^26^. Amylase is a digestive enzyme that catalyzes the hydrolysis of starch and is present in human saliva as well as in the pancreas. The human reference genome has three copies of the salivary amylase gene AMY1 and one copy each of the pancreatic amylase genes AMY2A and AMY2B. There is also a pseudogene AMYP1 containing a large part of the sequence of AMY2A. The copy numbers of amylase genes are highly variable, especially for AMY1, for which it varies from 2 to 22^27–29^. The copy number of AMY2A varies from 0 to 8, the least copy-variable is AMY2B with a copy number from 2 to 6. For testing the GeneToCN method, the *k*-mers were selected for each of these amylase genes and copy numbers were estimated from Illumina sequencing reads of 500 individuals from the Estonian Biobank (EstBB). Although the frequency of individual *k*-mers is variable, the consolidated information from all gene-specific *k*-mers allows reliable detection of differences between flanking region and gene region (Fig. 2).

**Figure 3:**
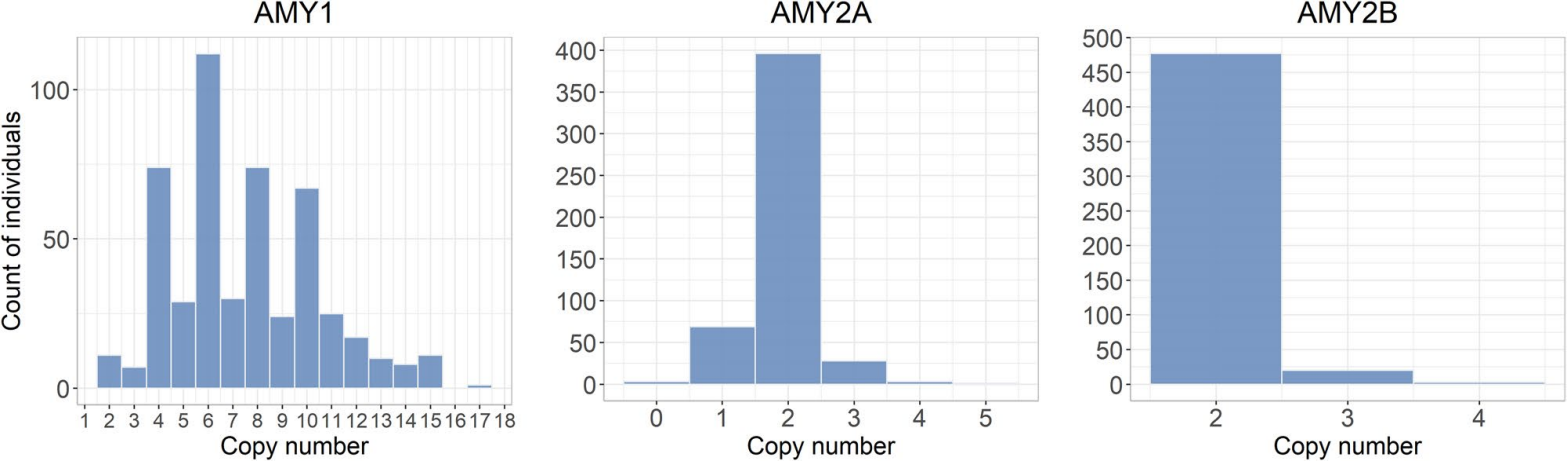
Amylase copy number distributions in 500 Estonian individuals from EstBB.

The distributions of predicted copy numbers in 500 individuals are shown in Fig. 3. As shown in previous studies^29, 30^, even copy numbers were more common than odd copy numbers for the AMY1 gene, with 73% of studied individuals having 4, 6, 8, or 10 copies. AMY2A copy numbers varied from 0 to 5 and AMY2B copy numbers varied from 2 to 4, which is also consistent with distributions observed in previous studies^29, 30^. For AMY2B we observed a duplication breakpoint within the first third of the gene at position Chr1:103,561,000 (Suppl. Figure S1). For those individuals copy number 4 was called by GeneToCN.

Previous studies on amylase gene copy numbers have shown that 98% of individuals have the same parity of the copy numbers of AMY1 and AMY2A, meaning that the copy numbers of these genes are usually both either even (more frequent) or both odd (rarely) at the same time^29^. GeneToCN predictions from 500 EstBB individuals (Fig. 3) showed the same parity in 85% of tested individuals, which is significantly higher parity than expected by chance alone (*P* = 1.253e^−14^), assuming that the AMY1 and AMY2A alleles are randomly paired.

### Experimental validation

**Figure 4:**
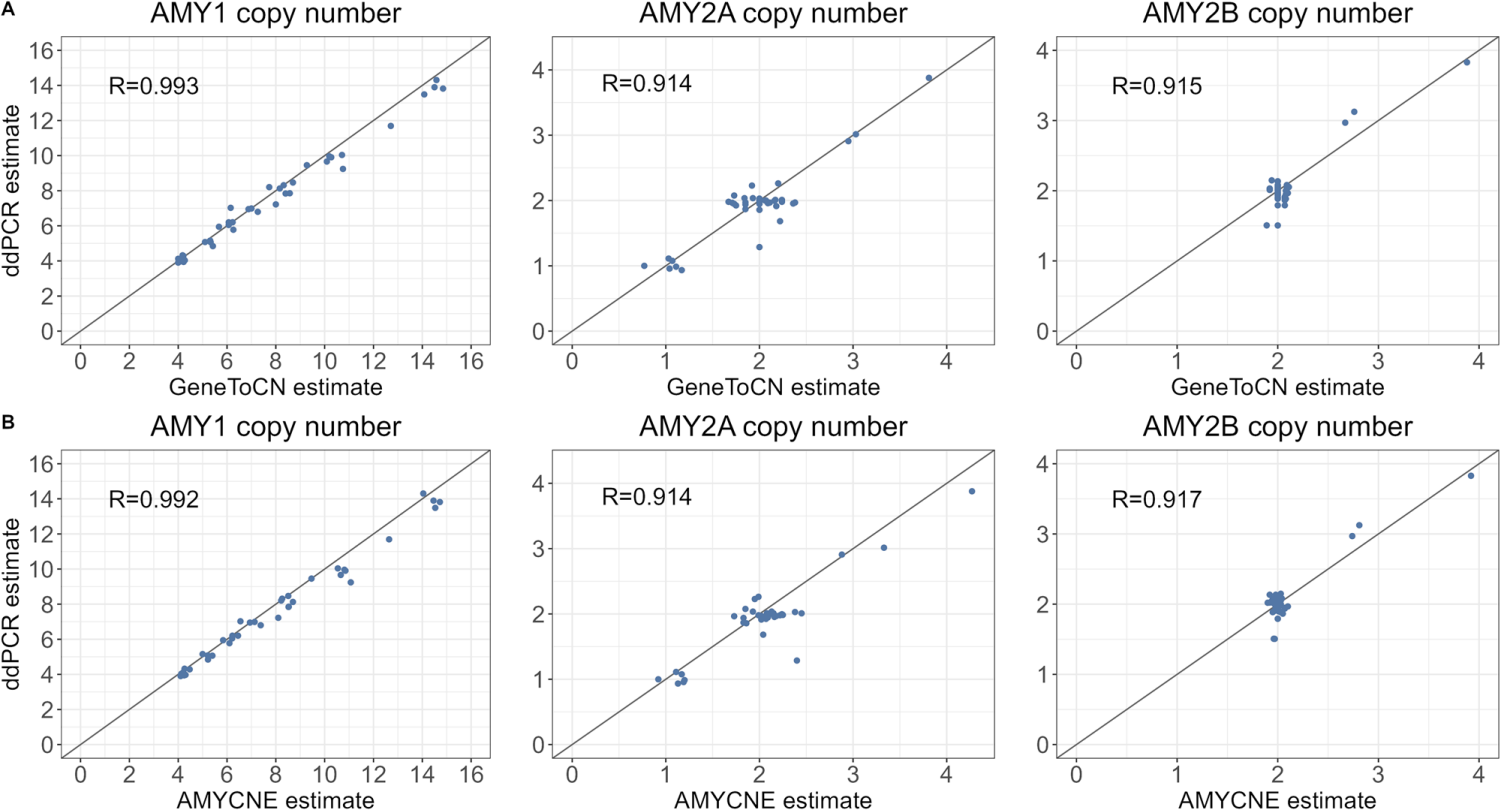
Correlation between copy number estimates from GeneToCN and ddPCR (**A**) and from AMYCNE and ddPCR (**B**) using 40 EstBB samples.

In addition to the analyses of frequency distributions (Fig. 3), the GeneToCN method was also validated experimentally using data from digital droplet PCR (ddPCR). For this, we used 40 individuals from EstBB, for which we had copy number data of AMY genes from previously published ddPCR experiments^29^. Although the experimental methods do not guarantee 100% accurate results, ddPCR has been used as the gold standard for experimental copy number determination^31, 32^ and is thus a good reference for the evaluation of the GeneToCN method. The correlation of copy number estimates from GeneToCN and ddPCR experiments for these 40 individuals are shown in Fig. 4A. Only one individual had a difference larger than 1 copy (8 copies according to ddPCR and 12 copies predicted by GeneToCN). We examined the *k*-mer frequency plot of this individual but could not detect any reasons that could explain the difference in predictions for this individual. All *k*-mers in the gene region support the prediction of 12 copies without any regional fluctuation, even at the locations of the ddPCR primers as shown in Supplementary Figure 2. Furthermore, the copy number estimated by AMYCNE, the read-depth based tool, was also 12. In light of these observations, we have reason to believe that the reported ddPCR copy number was likely incorrect, therefore, this data point was excluded from further calculations.

For numerical comparison, the correlation coefficient R was calculated from the raw results of both methods shown as a decimal number, whereas the concordance was calculated based on the integer copy number values (raw result rounded to the nearest integer). The results for all three amylase genes are shown in Supplementary Table S1. The correlation coefficient between predictions and experimental results was 0.99 for AMY1, 0.91 for AMY2A and 0.92 for AMY2B. The concordance was 74%, 97% and 100% for AMY1, AMY2A and AMY2B, respectively.

We observed that the correlation coefficient between GeneToCN and ddPCR predictions is 0.99, but the concordance of integer predictions is only 74% for AMY1 copy numbers. This is caused by the tendency of GeneToCN to slightly overestimate the copy number in individuals with > 8 copies of AMY1 (Fig. 4). Predictions could be improved, for example by using linear regression. We were able to increase the concordance of GeneToCN predictions to 85% by using the regression formula *y* = 0.9622*x for the correction. However, we did not implement this correction in the GeneToCN code because we do not have an independent dataset for testing the robustness of the correction on other gene regions.

### Comparison with AMYCNE

For comparison, the copy numbers of AMY genes in the same individuals were also estimated using a previously published software AMYCNE^17^. AMYCNE uses an algorithm based on read mapping and subsequent read depth analysis for copy number estimation. AMYCNE has been previously validated on amylase genes and would therefore be expected to be optimized for the analysis of these genes. In correlation analysis with ddPCR, we observed comparable accuracy for both GeneToCN and AMYCNE (Fig. 4B). For integer copy numbers, the predictions of AMY2A and AMY2B gene copy numbers were analogous, whereas GeneToCN predictions for the AMY1 gene had higher concordance with ddPCR results (Table S1). The parity of AMY1 and AMY2A copy number predictions in 39 individuals were 87%, 82% and 67% for ddPCR, GeneToCN and AMYCNE, respectively.

### Validation on FCGR3 genes

Copy numbers of FCGR3A and FCGR3B genes were estimated for 164 individuals using the low coverage whole genome sequencing data from 1000 Genome project^33^. The copy numbers for the same individuals have previously been estimated using AMYCNE and CNVnator^13^ and compared to the copy numbers that were determined using multiple different methods and therefore presumed to be the correct copy numbers^34^. Using the same truth set for comparison, the concordance of the copy number estimations for FCGR3A was 0.74 (0.71 and 0.5 from AMYCNE and CNVnator, respectively), while for FCGR3B, it was 0.63 (0.85 from both AMYCNE and CNVnator). Interestingly, even though GeneToCN yielded a higher concordance for FCGR3A, it did not demonstrate the same level of accuracy when estimating copy numbers for FCGR3B. In most cases (93% of the samples with inaccurate copy number estimate) the copy number was underestimated. Looking at the *k*-mer frequency plots, we noticed that a subset of k-mers frequently exhibited unexpectedly low frequencies. Therefore, this *k*-mer database could potentially benefit from the implementation of an additional filtering mechanism.

### Testing on different gene regions

We tested GeneToCN thoroughly on the amylase gene region as well as FCGR3 genes. However, it would be important to know if the same method can be used for the estimation of copy numbers of other genes, particularly whether a sufficient number of *k*-mers can be selected from gene regions and flanking regions. We created custom *k*-mer databases for a set of genes from different genomic regions and with different copy numbers: survival of motor neuron genes SMN1 and SMN2^35–37^, the human pancreatic polypeptide receptor gene NPY4R^38, 39^, and the LPA gene. In the latter case, the repeated region consists of only one protein domain, the 5.5 kb long Kringle-IV type 2 domain, which spans over 2 exons^40, 41^. This case allowed us to validate the suitability of the method not only on full genes but on shorter high-copy repeats as well. For these genes, we tested whether an adequate number of *k*-mers can be selected and whether their distributions of predicted copy numbers coincide with previously published copy number distributions.

**Figure 5:**
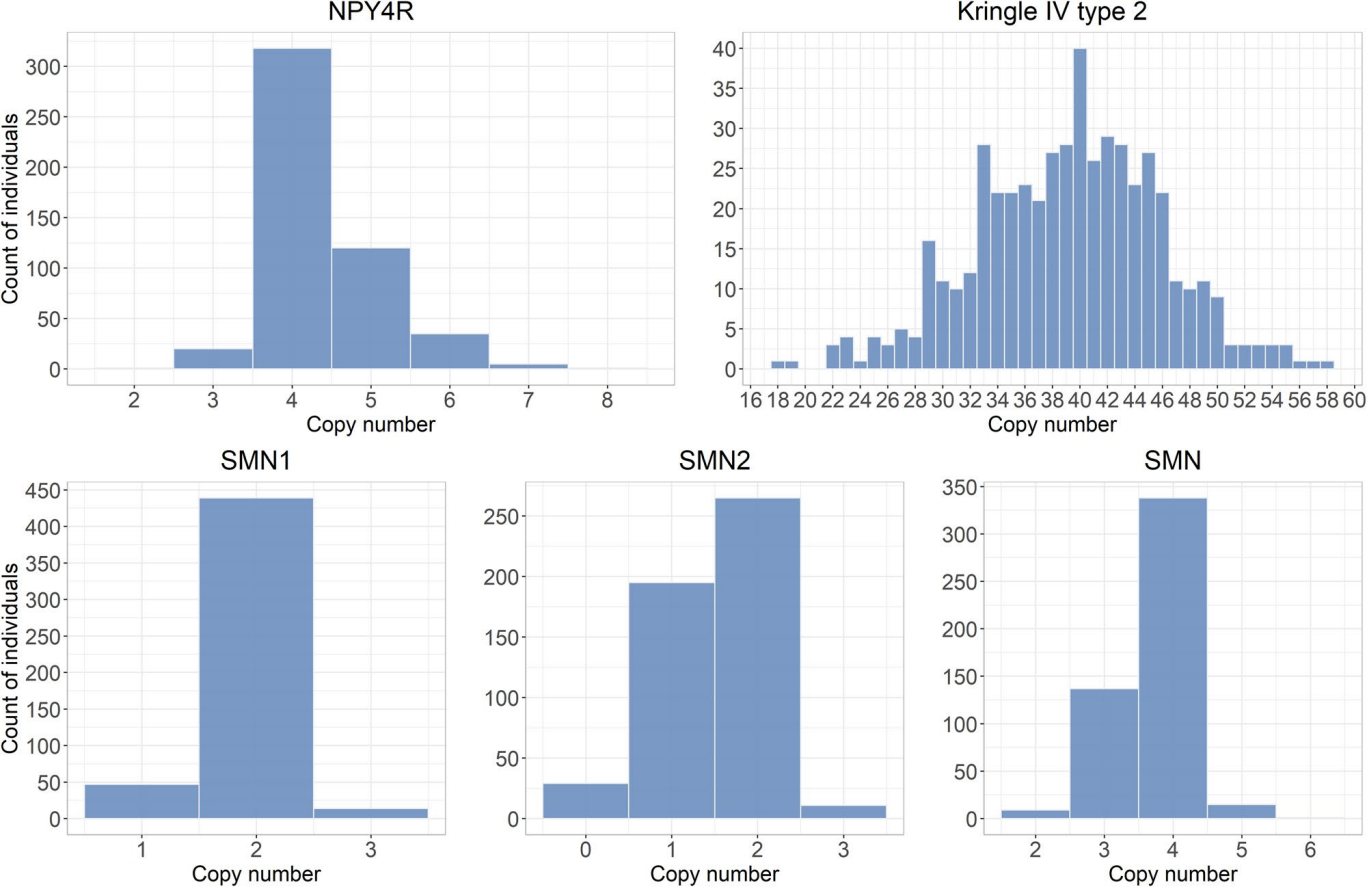
Copy number distributions for 500 individuals, estimated using GeneToCN. Panel SMN represents the sum of SMN1 and SMN2 gene copies.

For each of these genes, *k*-mers were selected with GeneToKmer, and copy numbers were estimated with the KmerToCN tool from the 500 EstBB individuals as described above. The distributions of predicted copy numbers are shown in Fig. 5. The copy numbers for the NPY4R gene varied from 2 to 8 and the most common copy number was 4. The copy numbers for SMN1 varied between 1 and 3, whereas for the SMN2 gene, the copy number estimates were between 0 and 3. For the Kringle-IV type 2 domain, the copy numbers were between 18 and 58 (mean 39.2), with the most common copy number being 40. In a previous study, where copy numbers of the Kringle-IV type 2 region were estimated with the Genome STRiP for a larger sample of 2284 Estonians from the EstBB, the copy numbers varied between 12 and 63 with a mean of 39.7^42^. These results, particularly the fact that mean copy numbers of the Kringle-IV type 2 domain are very similar, confirm that the GeneToCN method is robust and usable for the estimation of copy numbers for even high-copy repeats.

Analysis of SMN1 and SMN2 genes revealed some limitations of the GeneToCN method. These two genes have a nucleotide-level identity of about 99.9%, therefore only a limited number of gene-specific *k*-mers (268 and 267, respectively) could be selected. It is not clear how accurately copy numbers are inferred from such a small number of *k*-mers. To better evaluate the accuracy of SMN1 and SMN2 copy numbers, a separate *k*-mer database was created for estimating the total number of SMN copies, using the *k*-mers present in both genes. For 77% of the individuals, the sum of SMN1 and SMN2 copy numbers estimated separately matched the total SMN copy number. The 500 individuals were then divided into two groups based on whether the sum matched the total SMN copy number or not. We observed that the group where the copy numbers did not match had significantly lower (Wilcoxon test, *P* = 2.2*10^–16^) copy number values for SMN2, as well as for SMN1 (*P* = 0.0023). This can be explained by single nucleotide variants in the SMN genes that may cause underestimation of the SMN2 and in some cases SMN1 copy number. Overall, it seems that the number of gene-specific *k*-mers in SMN1 and SMN2 is too small to allow reliable estimation of their copy numbers separately. However, both SMN genes together had > 16,000 gene-specific *k*-mers allowing reliable prediction of their cumulative copy number.

### Copy numbers estimated from long-read sequencing data

Long-read sequencing data from Oxford Nanopore and PacBio sequencing technologies were used in addition to Illumina reads to evaluate how the method works on other sequencing data apart from Illumina. The comparisons were done on a reference sample CHM13 that has been sequenced by three different technologies^43^ to 50x (Illumina), 120x (Oxford Nanopore), and 30x (PacBio) depth of coverage. The copy numbers were estimated for eight gene regions and the results are shown in Supplementary Table S2. Overall, the copy number predictions are similar with all three technologies.

**Figure 6:**
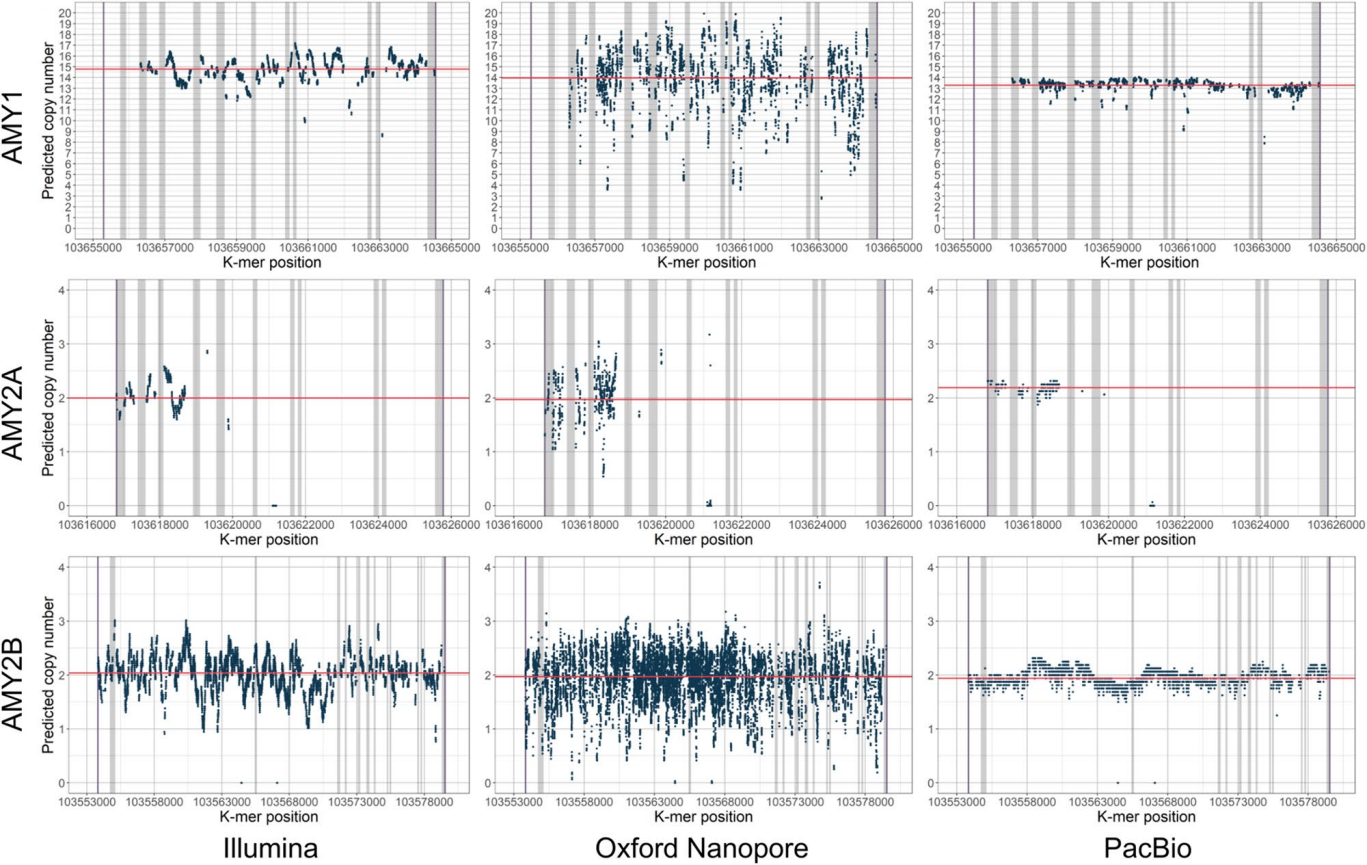
Normalized *k*-mer frequencies in AMY1, AMY2A, and AMY2B regions from sequencing data generated by Illumina, Oxford Nanopore, and PacBio technologies. The *x*-axis shows the *k*-mer locations on chromosome 1. The horizontal red line marks the copy number estimated by GeneToCN. Exon regions are shown in grey.

A visual overview of *k*-mer frequency variation from AMY1, AMY2A, and AMY2B regions is shown in Fig. 6, and NPY4R, SMN, LPA Kringle IV-2 and FCGR3 regions are shown in Supplementary Figure S3 and in Supplementary Figure S4. We observed a difference in variations of *k*-mer frequencies between the three technologies. The Oxford Nanopore data is affected by the high mutation rate, resulting in high variability. PacBio data is the least variable. However, these differences do not have any systematic adverse effects on the copy number estimation, making us conclude that all three technologies are suitable for the alignment-free inference of copy numbers.

## Methods

### Creation of *k*-mer databases

The *k*-mer databases for gene regions were compiled using the GeneToKmer program that utilizes tools from version 4.2.16 of the GenomeTester4 package from GitHub^20^. The *k*-mer length used throughout this study was 25. The coordinates used for each region can be seen in Supplementary Table S3. The first step of the *k*-mer selection process is creating a set of overlapping *k*-mers for each gene region. These *k*-mers were generated by a moving window using the GListMaker and human reference genome GRCh38p10. For regions that had multiple copies in the reference genome (for example Kringle IV type 2 with 6 copies or AMY1 with 3 copies), separate *k*-mer lists were initially created for each region and then the intersect of the common *k*-mers was taken.

For the selection of region-specific and unique *k*-mers, the frequencies of all the *k*-mers were then counted from the reference genome. For this, we used a total *k*-mer list compiled from the GRCh38p10 reference genome. The frequencies of all *k*-mers in the genome were obtained using the tool GListQuery from GenomeTester4. *K*-mers were considered unique and gene-specific when the frequency in the reference genome was equal to the number of copies of that gene in the reference (for example 3 for AMY1). We used the 1 mismatch (-mm 1) option to exclude any *k*-mers that had identical *k*-mers or *k*-mers with edit distance 1 present somewhere else in the reference genome (except for SMN1 and SMN2 regions where we did not use that option). The region-specific *k*-mers were further filtered by GC-content. We filtered out all *k*-mers with a GC-content value lower than 20 or higher than 65.

It is possible to use GeneToKmer in the same manner for compiling databases for flanking *k*-mers. However, in regions with repeated content, the flanking region might need to be rather large (several million base pairs) and finding unique *k*-mers from these regions might require lengthy calculations. Alternatively, a reference region with a non-variable copy number may be chosen further away from the gene.

In this project, we decided to use subsets of unique *k*-mers previously compiled for NIPTmer prenatal diagnostic software^44^. As CHM13 assembly was used in this project, NIPTmer *k*-mers were further filtered based on the uniqueness of these *k*-mers in the CHM13 assembly. In the initial database, the *k*-mers were divided into groups based on their locations, the exact coordinates of the *k*-mers were unspecified. We chose the groups that were closest to the genes of interest, the coordinates of used regions and the number of selected gene-specific and flanking *k*-mers are shown in Supplementary Table S3. However, an updated version of this database is now available on GeneToCN GitHub page, where the coordinates are provided for each separate *k*-mer. For each gene, we recommend utilizing a minimum of 2000 flanking *k*-mers closest to the gene, evenly divided between the 5' and 3' flanking region.

### Copy number estimation

Copy number estimations were done with the KmerToCN software script, which uses either gmer_counter from the FastGT toolkit^22^ or optionally GListMaker and GListQuery from the GenomeTester4 toolkit^20^ for *k*-mer counting, depending on the input file type of the sequencing data (FASTQ or *k*-mer list).

AMYCNE required running another tool called TIDDIT^45^, both tools are written in Python. AMYCNE needed some modifications in the code as well as in several input files to work. For AMY2A, the sequence coordinates proposed by the authors were altered by keeping only the first part of the gene up until the fourth exon (since the rest of the gene sequence is identical to the sequence of the pseudogene AMYP1), which improved the overall correlation with ddPCR results from 0.54 to 0.92 and integer copy number concordance from 0.58 to 0.98.

The ddPCR copy numbers used for validation of the method and the process of ddPCR experiments were described in a previous study^29^.

### Sequencing data

Method validation and copy number estimations were conducted using 500 samples from the Estonian Biobank, of which 40 samples were used also for the comparison of AMY1, AMY2A, and AMY2B copy numbers estimated by different methods. The Illumina sequencing data (ca 30x depth of coverage, read length of 151 bp) for the Estonian Biobank samples were retrieved from the Estonian Genome Centre. For the validation on FCGR3 genes, low coverage sequencing data of 164 individuals was obtained from 1000 Genomes Project^33^. The Illumina, Oxford Nanopore, and PacBio sequencing data for CHM13^43^ were retrieved from https://github.com/marbl/CHM13/blob/master/Sequencing_data.md.

### Computational performance

Creating the *k*-mer databases for a gene region with GeneToKmer typically takes less than 15 min. The time usage depends mostly on the length of the gene regions. We measured 105 s of user CPU time for creating *k*-mer databases for three gene regions with a total length of 36,000 bp, and 5 Mb as peak memory usage.

The time usage of KmerToCN, including counting the* k*-mers from the FASTQ files and estimating the copy numbers, depends on the number and sizes of input FASTQ files and is similar to the speed we have demonstrated previously for other alignment-free genome analysis tools^22, 24^. In this study we measured an average user CPU time of 7621 s and real elapsed time of 32 min using low coverage sequencing data, and a peak memory usage of 12.6 Mb. A comparison with AMYCNE (measured using the same sequencing data) can be seen in Supplementary Table S4. The performance was measured on a Linux server with 64 CPUs (2.27 GHz) and 512 GB RAM.

## Discussion

In this study, we propose a novel alignment-free method GeneToCN for targeted gene copy number estimation. In this approach, we use local *k*-mer frequencies from the flanking regions of a gene as a reference for normalization. Defining the "flanking regions" for *k*-mer selection assumes that we know the approximate breakpoints of the copy-variable region, which in most cases are already available from previous studies. Alternatively, *k*-mers from other known non-copy-variable regions, preferably located near the targeted genes, can be used. Novel breakpoints can be detected from *k*-mer frequency plots of each individual.

What are the advantages of using raw sequencing reads instead of mapped reads for variant detection, particularly for gene copy number estimation? The sequencing data are often stored in a BAM or CRAM format where reads are already mapped to the reference genome. However, there are some important benefits for variant detection and copy number estimation directly from raw sequencing reads. Most importantly, using the raw data makes the method more robust and easy to use. An alignment-free method averts the effect of methodological errors in read mapping (due to mismapping or incorrect reference sequence) which simplifies the analysis process and may increase the accuracy of the results in some regions. Also, speed and the consequent decrease in computational costs are beneficial in large-scale studies where thousands of individuals need to be analyzed. For example, in a meta-analysis of large datasets, it is necessary to use the same analysis pipeline for all individuals. In this case, it might not be practical to re-map all the reads in meta-analysis projects, but rather use a fast alignment-free approach.

What is the minimum depth of coverage for alignment-free analysis? This is a complicated question without an easy answer. There is an interplay between the depth of coverage and number of *k*-mers in the region. It is assumed that a higher overall number (either because of sequencing depth or from region width) of *k*-mers would give more accurate predictions. Another factor is how equally the *k*-mers are distributed over the gene region. A closely located (or overlapping) set of *k*-mers is more prone to be influenced by local fluctuations in frequency and therefore less reliable. In a previous study, we conducted an in-depth analysis suggesting that 20 is the minimum required depth of coverage for alignment-free genotyping of single nucleotide variants^22^. For copy number predictions we use hundreds or thousands of *k*-mers (for example 3095 for AMY1), therefore a smaller depth of coverage might be sufficient. For instance, we saw that the accuracy of FCGR3A copy number estimations from low coverage sequencing data was higher using GeneToCN, compared to the results from AMYCNE and CNVnator. However, the exact limits of the method and correlation between the number of *k*-mers per region, depth of coverage and accuracy of copy number predictions need further investigation. In any case, regardless of the high accuracy that was achieved for FCGR3A from low coverage data, we would advise to use sequencing data with higher coverage, if possible, as the accuracy of copy number estimations is ultimately dependent on the quality of the data utilized.

How to explain the differences between the two computational methods GeneToCN and AMYCNE^17^? In most analyses, they demonstrate very similar performance. For example, their correlation with experimental ddPCR predictions was nearly identical (Table S1). The difference appeared only in copy number prediction of the AMY1 gene, which has up to 16 copies. For the AMY1 region, GeneToCN had higher concordance with ddPCR results (74% vs 67%) and a higher level of parity between AMY1 and AMY2A copy numbers (82% vs 67%) compared to AMYCNE. This difference in high copy number predictions could appear from the different approaches we use for filtering *k*-mers in regions where a gene is represented in multiple copies in the reference genome.

As a further development of GeneToCN, we plan to compile and publish *k*-mer databases for all genes that are copy-variable or contain smaller copy-variable regions of interest. This would provide users with an easily accessible toolbox for alignment-free copy-number prediction.

### Supplementary Information

Supplementary Information.

## Data Availability

The source code and *k*-mer databases for analyzed genes are available on GitHub (https://github.com/bioinfo-ut/GeneToCN). The binaries and source code of the *k*-mer counting software GenomeTester4 are available on GitHub (https://github.com/bioinfo-ut/GenomeTester4/). GenomeTester4 is distributed under the terms of GNU GPL v3, and the *k*-mer databases are distributed under the Creative Commons CC BY-NC-SA license.

## References

[1] Sebat J (2004). Large-scale copy number polymorphism in the human genome. Science.

[2] Feuk L, Carson AR, Scherer SW (2006). Structural variation in the human genome. Nat. Rev. Genet..

[3] Zarrei M, MacDonald JR, Merico D, Scherer SW (2015). A copy number variation map of the human genome. Nat. Rev. Genet..

[4] Kosugi S (2019). Comprehensive evaluation of structural variation detection algorithms for whole genome sequencing. Genome Biol..

[5] Collins RL (2020). A structural variation reference for medical and population genetics. Nature.

[6] Pös O (2021). DNA copy number variation: Main characteristics, evolutionary significance, and pathological aspects. Biomed. J..

[7] Conrad DF (2010). Origins and functional impact of copy number variation in the human genome. Nature.

[8] Beckmann JS, Estivill X, Antonarakis SE (2007). Copy number variants and genetic traits: closer to the resolution of phenotypic to genotypic variability. Nat. Rev. Genet..

[9] Weischenfeldt J, Symmons O, Spitz F, Korbel JO (2013). Phenotypic impact of genomic structural variation: Insights from and for human disease. Nat. Rev. Genet..

[10] Sudmant PH (2010). Diversity of human copy number variation and multicopy genes. Science.

[11] Korbel JO (2007). Paired-end mapping reveals extensive structural variation in the human genome. Science.

[12] Medvedev P, Fiume M, Dzamba M, Smith T, Brudno M (2010). Detecting copy number variation with mated short reads. Genome Res..

[13] Abyzov A, Urban AE, Snyder M, Gerstein M (2011). CNVnator: An approach to discover, genotype, and characterize typical and atypical CNVs from family and population genome sequencing. Genome Res..

[14] Miller CA, Hampton O, Coarfa C, Milosavljevic A (2011). ReadDepth: A parallel R package for detecting copy number alterations from short sequencing reads. PLOS ONE.

[15] Handsaker RE, Korn JM, Nemesh J, McCarroll SA (2011). Discovery and genotyping of genome structural polymorphism by sequencing on a population scale. Nat Genet.

[16] Handsaker RE (2015). Large multiallelic copy number variations in humans. Nat. Genet.

[17] Eisfeldt J, Nilsson D, Andersson-Assarsson JC, Lindstrand A (2018). AMYCNE: Confident copy number assessment using whole genome sequencing data. PLoS One.

[18] Marçais G, Kingsford C (2011). A fast, lock-free approach for efficient parallel counting of occurrences of k-mers. Bioinformatics.

[19] Wood DE, Salzberg SL (2014). Kraken: ultrafast metagenomic sequence classification using exact alignments. Genome Biol..

[20] Kaplinski L, Lepamets M, Remm M (2015). GenomeTester4: a toolkit for performing basic set operations - union, intersection and complement on k-mer lists. Gigascience.

[21] Audano PA, Ravishankar S, Vannberg FO (2018). Mapping-free variant calling using haplotype reconstruction from k-mer frequencies. Bioinformatics.

[22] Pajuste F-D (2017). FastGT: An alignment-free method for calling common SNVs directly from raw sequencing reads. Sci Rep.

[23] Kaplinski L, Möls M, Puurand T, Pajuste F-D, Remm M (2021). KATK: Fast genotyping of rare variants directly from unmapped sequencing reads. Human Mutat..

[24] Puurand T, Kukuškina V, Pajuste F-D, Remm M (2019). AluMine: alignment-free method for the discovery of polymorphic Alu element insertions. Mob DNA.

[25] Shen F, Kidd JMR (2020). Paralog-sensitive CNV analysis of 2457 human genomes using quicK-mer2. Genes.

[26] Groot PC (1989). The human alpha-amylase multigene family consists of haplotypes with variable numbers of genes. Genomics.

[27] Groot PC, Mager WH, Frants RR (1991). Interpretation of polymorphic DNA patterns in the human alpha-amylase multigene family. Genomics.

[28] Perry GH (2007). Diet and the evolution of human amylase gene copy number variation. Nat. Genet..

[29] Usher CL (2015). Structural forms of the human amylase locus and their relationships to SNPs, haplotypes and obesity. Nat. Genet..

[30] Inchley CE (2016). Selective sweep on human amylase genes postdates the split with Neanderthals. Sci. Rep..

[31] Hindson CM (2013). Absolute quantification by droplet digital PCR versus analog real-time PCR. Nat. Methods.

[32] Härmälä SK, Butcher R, Roberts CH (2017). Copy number variation analysis by droplet digital PCR. Methods Mol. Biol..

[33] Auton A, Brooks LD, Durbin RM, Garrison EP, Kang HM, Consortium GP (2015). A global reference for human genetic variation. Nature..

[34] Qi YY (2016). Comparison of multiple methods for determination of FCGR3A/B genomic copy numbers in HapMap asian populations with two public databases. Front. Genet..

[35] Rochette CF, Gilbert N, Simard LR (2001). SMN gene duplication and the emergence of the SMN2 gene occurred in distinct hominids: SMN2 is unique to Homo sapiens. Hum. Genet..

[36] Schmutz J (2004). The DNA sequence and comparative analysis of human chromosome 5. Nature.

[37] Butchbach MER (2021). Genomic variability in the survival motor neuron genes (SMN1 and SMN2): Implications for spinal muscular atrophy phenotype and therapeutics development. Int. J. Mol. Sci..

[38] Jarick I (2011). Novel common copy number variation for early onset extreme obesity on chromosome 11q11 identified by a genome-wide analysis. Hum. Mol. Genet..

[39] Shebanits K (2018). Copy number of pancreatic polypeptide receptor gene NPY4R correlates with body mass index and waist circumference. PLoS One.

[40] Lanktree MB, Anand SS, Yusuf S, Hegele RA (2010). Share investigators. comprehensive analysis of genomic variation in the LPA locus and its relationship to plasma lipoprotein(a) in South Asians, Chinese, and European Caucasians. Circ. Cardiovasc. Genet..

[41] Noureen A, Fresser F, Utermann G, Schmidt K (2015). Sequence variation within the KIV-2 copy number polymorphism of the human LPA gene in African, Asian, and European populations. PLoS One.

[42] Zekavat SM (2018). Deep coverage whole genome sequences and plasma lipoprotein(a) in individuals of European and African ancestries. Nat. Commun..

[43] Nurk S (2022). The complete sequence of a human genome. Science.

[44] Sauk M (2018). NIPTmer: Rapid k-mer-based software package for detection of fetal aneuploidies. Sci. Rep..

[45] J. Eisfeldt, F. Vezzi, P. Olason, D. Nilsson & A. Lindstrand TIDDIT, An efficient and comprehensive structural variant caller for massive parallel sequencing data. F1000Res **6**, 664 (2017) 10.12688/f1000research.11168.1 PMC5521161 28781756

