# Supplementary material for: GeneToCN: an alignment-free method for gene copy number estimation directly from next-generation sequencing reads
Source: Sci Rep. 2023 Oct 18;13:17765. doi: 10.1038/s41598-023-44636-z (PMC10584998; doi:10.1038/s41598-023-44636-z)
Supplement: Supplementary file 1 — Supplementary Information. [file 41598_2023_44636_MOESM1_ESM.pdf]

GeneToCN: an alignment-free method for gene copy number estimation directly from next-generation sequencing reads

Fanny-Dhelia Pajuste, Maido Remm

SUPPLEMENTARY DATA

Table S1. Comparison of the results from GeneToCN and from AMYCNE to the experimental results from digital droplet PCR

	Correlation coefficient R		*Concordance*	
	GeneToCN	AMYCNE	*GeneToCN*	AMYCNE
AMY1	0.993	0.992	74.4%	66.7%
AMY2A	0.914	0.914	97.4%	97.4%
AMY2B	0.915	0.917	100%	100%

* This result was achieved after manually removing the part that is repeated in pseudogene AMYP1. Without the removal of the problematic region, the AMYCNE gave a correlation coefficient of R=0.54 and a concordance of 58%.

Table S2. Copy numbers of different genes estimated for the CHM13 cell line from Illumina, Oxford Nanopore, and PacBio sequencing data.

Gene region	*Illumina*	*Nanopore*	*PacBio*
AMY1	14.8	14.0	13.3
AMY2A	2.0	2.0	2.2
AMY2B	2.0	2.0	2.0
NPY4R	3.8	4.6	4.5
SMN	3.9	4.4	3.7
LPA Kringle IV-2	45.2	39.9	48.7
FCGR3A	1.93	1.6	2.0
FCGR3B	3.41	2.8	3.93

Table S3. The coordinates of gene-specific regions for *k*-mer selection, used in this study. The reference genome was GRCh38p10.

Region	Chr	Region coordinates	Number of gene region *k*-mers	Flanking region coordinates	Number of flanking region *k*-mers
AMY1	1	103,655,290 – 103,664,554<br>103,687,415 – 103,696,680<br>103,750,406 – 103,758,690	3095	103,305,000 - 104,305,000	1875
AMY2A	1	103,616,811 – 103,625,780	738		
AMY2B	1	103,553,815 – 103,579,534	14764		
NPY4R	10	46,461,099 – 46,465,958<br>47,918,662 – 47,923,524	4042	46,781,000 - 48,474,000	2099
LPA Kringle IV-2	6	160,639,460 – 160,645,006<br>160,633,913 – 160,639,459<br>160,628,367 – 160,633,912<br>160,622,823 – 160,628,366<br>160,617,277 – 160,622,822<br>160,611,722 – 160,617,276	1781	160,647,000 - 161,617,000	2456
SMN1	5	70,925,030 - 70,953,942	268	69,210,000 - 69,534,000<br>71,324,000 - 72,211,000	2633
SMN2	5	70,049,638 - 70,078,522	267		
SMN	5	70,925,030 - 70,953,942<br>70,049,638 - 70,078,522	16273		
FCGR3A	1	161,541,759-161,550,968	1038	179,365,585 - 180,365,114	2417
FCGR3B	1	161,623,196-161,631,963	998		

Table S4. Time (the amount of user CPU time) and peak memory usage for copy number estimation from raw sequencing data. KmerToCN includes all the steps to get copy number estimations based on FASTQ files, including *k*-mer counting. The steps that are not required for every run (for example creating k-mer databases with GeneToKmer or creating GC content file for AMYCNE) are not included.

	Step	*Time (s)*	*Memory (Mb)*
AMYCNE	Bowtie2 + samtools	243856	3.9
	TIDDIT	649	0.3
	AMYCNE	330	2.2
	Total:	244505	3.9
GeneToCN	KmerToCN	7621	12.6

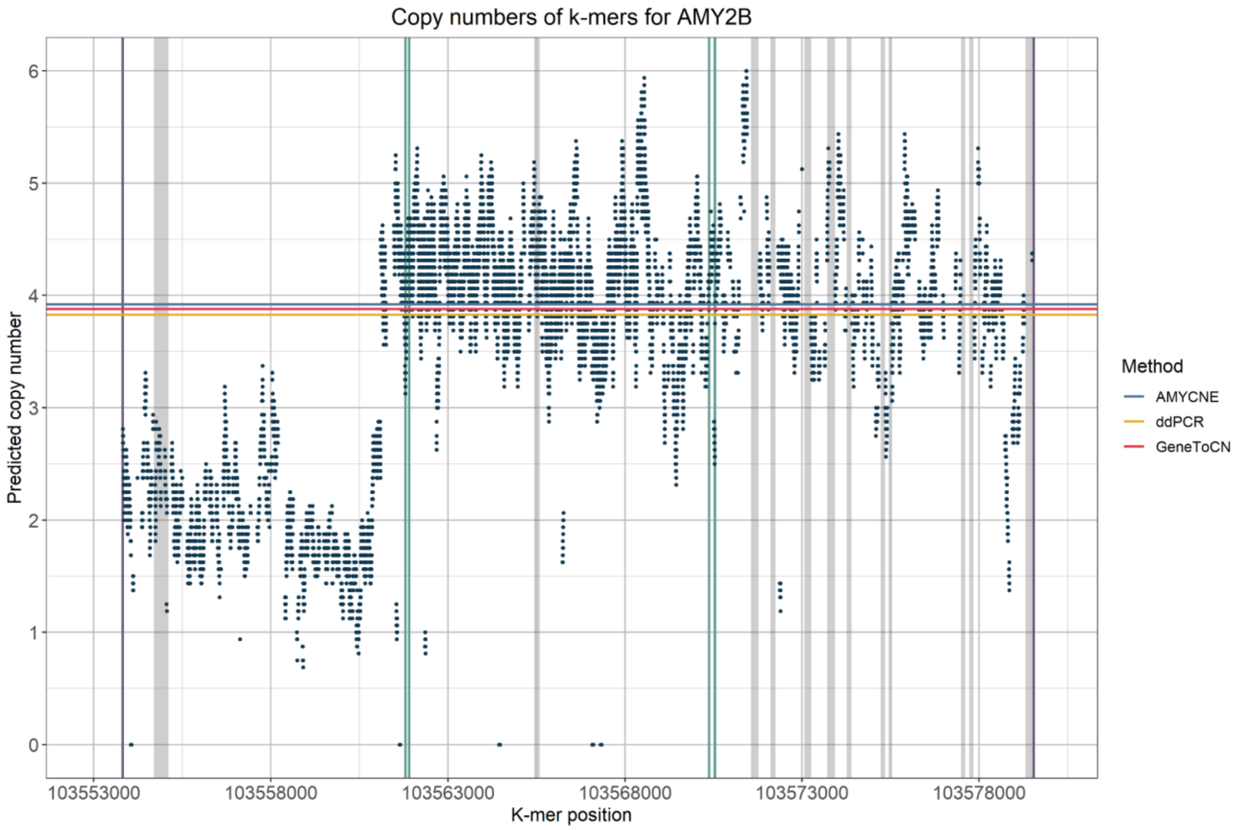

Figure S1. An example of atypical copy number change within the gene (atypical breakpoint). The x-axis shows the *k*-mer location in chromosome 1. The horizontal red line marks the average copy number estimated by KmerToCN. Green lines denote the locations of ddPCR primers.

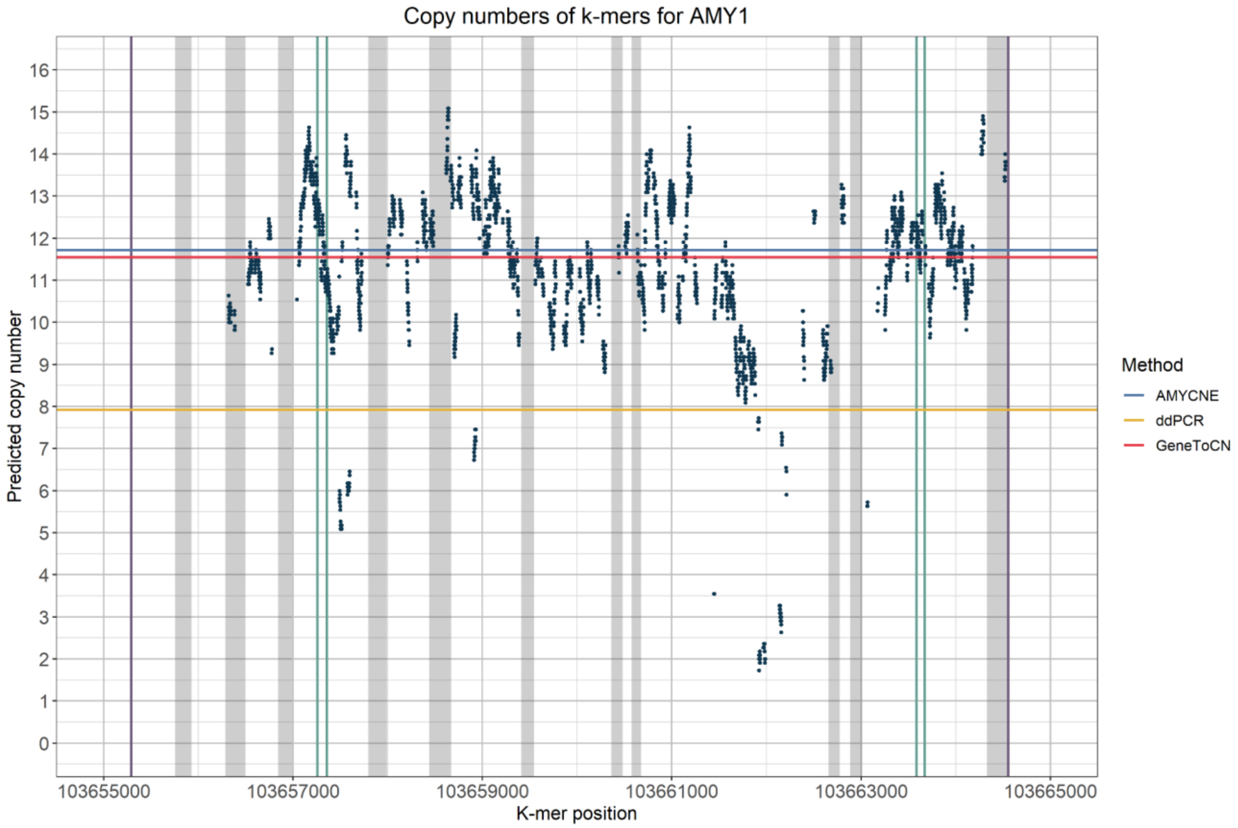

Figure S2. An individual with a large difference between ddPCR and GeneToCN predictions. Green vertical lines indicate the locations of ddPCR primer pairs. We can observe that *k*-mer frequencies in PCR primer regions that influence the ddPCR predictions have no obvious differences from the copy number estimated by GeneToCN and AMYCNE.

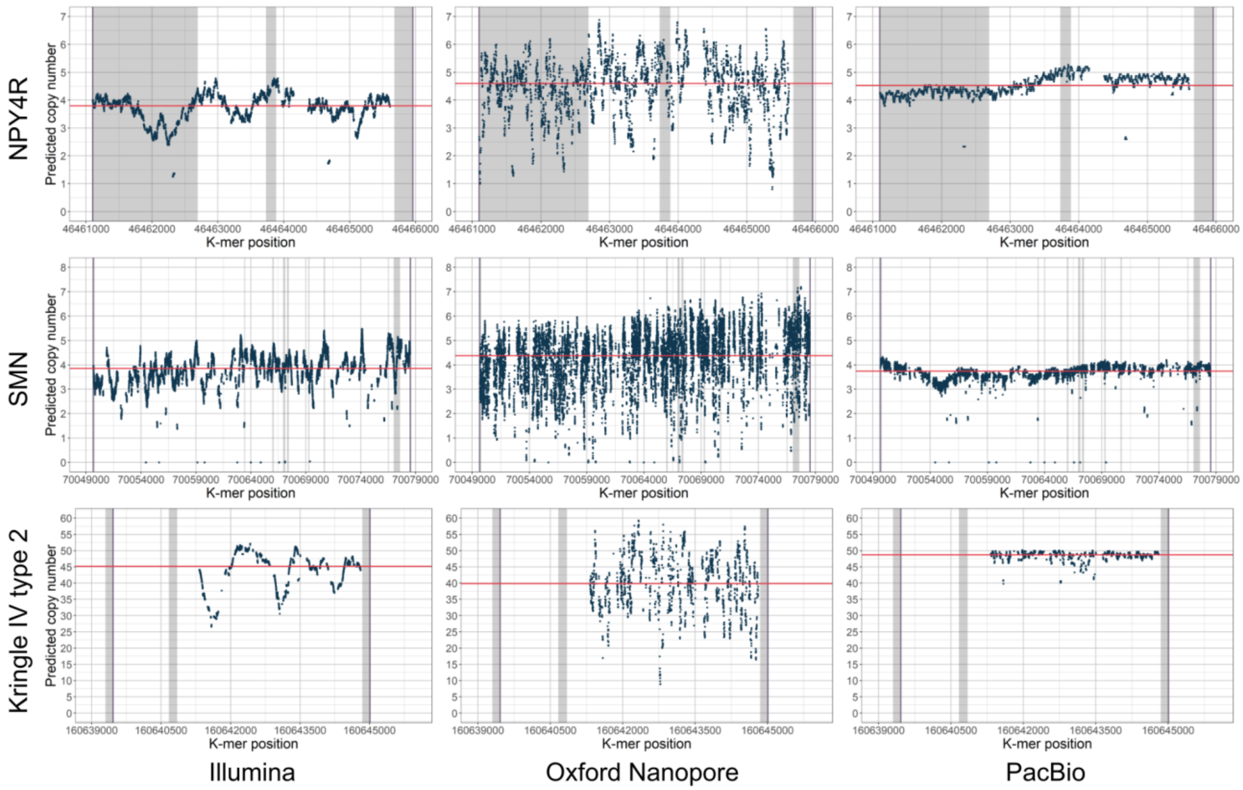

Figure S3. *K*-mer frequencies in NPY4R, SMN, and LPA Kringle IV regions from sequencing data generated by Illumina, Oxford Nanopore, and PacBio technologies. The x-axis shows the *k*-mer locations on the respective chromosomes. The horizontal red line marks the copy number estimated by GeneToCN. Exon regions are shown in grey.

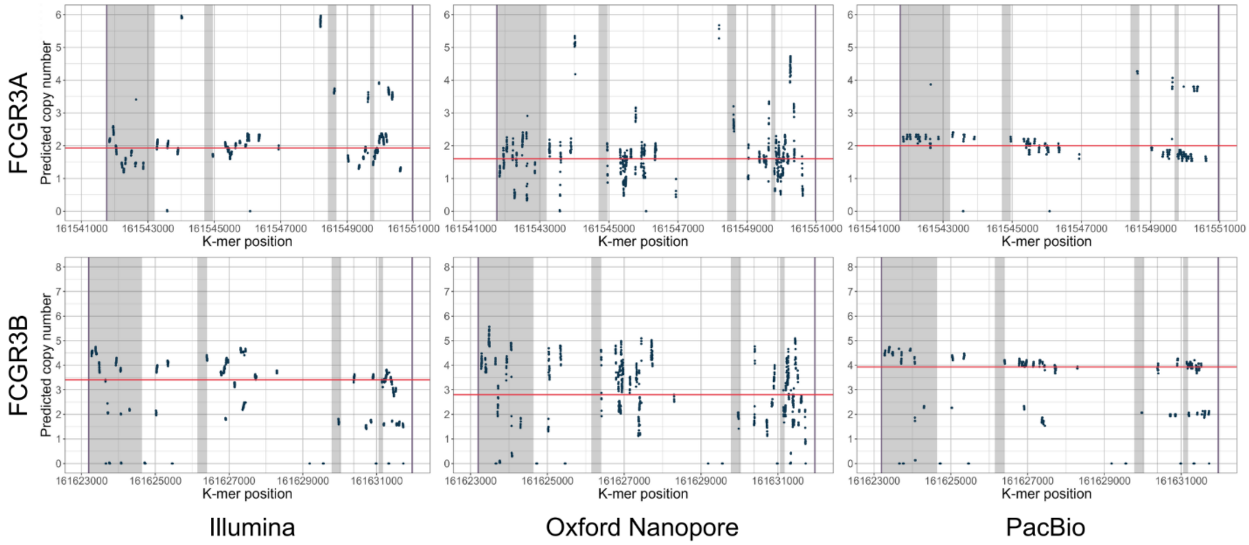

Figure S4. *K*-mer frequencies in FCGR3A and FCGR3B regions from sequencing data generated by Illumina, Oxford Nanopore, and PacBio technologies. The x-axis shows the *k*-mer locations on chromosome 1. The horizontal red line marks the copy number estimated by GeneToCN. Exon regions are shown in grey.

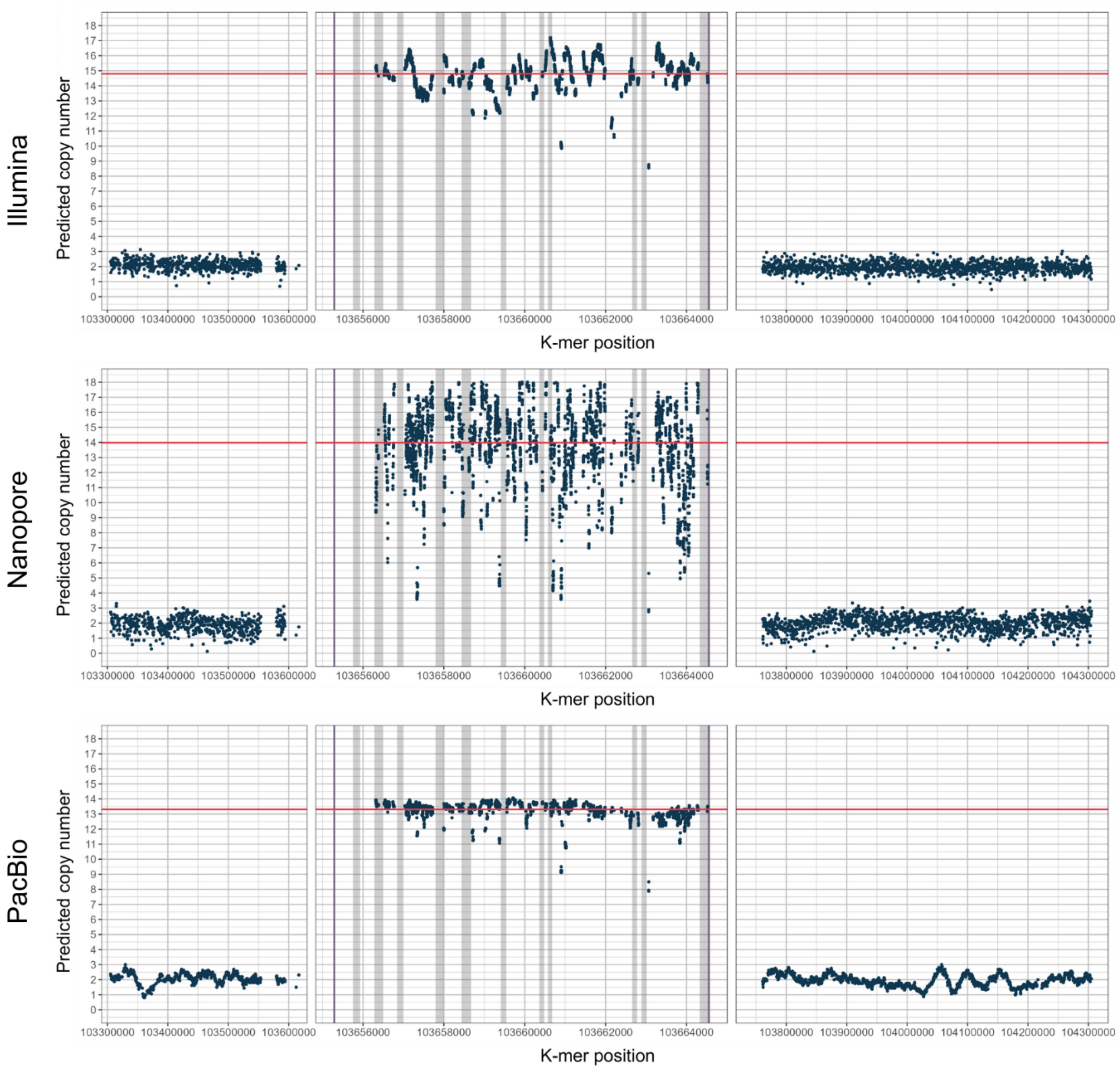

Figure S5. Normalized *k*-mer frequencies in AMY1 region from sequencing data generated by Illumina, Oxford Nanopore, and PacBio technologies. The x-axis shows the *k*-mer locations on chromosome 1. The horizontal red line marks the copy number estimated by GeneToCN. Each panel shows a 5'-flanking region, a zoomed-in AMY1 gene region, and a 3'-flanking region. Exon regions are shown in grey.
